# Pharmacological Management of Oral and Esophageal Candidiasis: A Clinical Pharmacotherapy Perspective

**DOI:** 10.3390/jcm14217537

**Published:** 2025-10-24

**Authors:** Toshinori Hirai, Masanori Nashi

**Affiliations:** 1 Department of Pharmacy, Institute of Science Tokyo Hospital, 1-5-45 Yushima, Bunkyo-ku 113-8519, Tokyo, Japan; 2 Department of Oral and Maxillofacial Surgery, Nishinomiya Watanabe Hospital, 10-22 Murokawa-cho, Nishinomiya 662-0863, Hyogo, Japan; m-nashi@n-watanabe-hosp.jp

**Keywords:** Candida, antifungals, pharmacotherapy, toxicity, drug–drug interactions

## Abstract

*Candida* spp. are common components of normal microbiota in the oral cavity. However, Candida albicans can be a primary cause of superficial infections in the oral cavity and esophagus, especially in immunocompromised individuals. While these infections are rarely life-threatening, they can significantly impair quality of life and, in severe cases, progress to hematogenous dissemination. Oral candidiasis often exhibits as pseudomembranous, erythematous (atrophic), chronic hyperplastic, denture stomatitis, or angular cheilitis. Esophageal candidiasis is typically diagnosed by upper endoscopy, which involves histological examination and brushing. Clinical guidelines recommend topical antifungal agents for mild oral candidiasis, and systemic agents for moderate-to-severe disease or when topical therapy fails. However, azole antifungals pose a substantial risk of drug–drug interactions, primarily due to the inhibition of cytochrome P450 enzymes and drug transporters, which dramatically alters the pharmacokinetics of co-administered drugs. Additionally, amphotericin B, a polyene macrolide antibiotic, may cause nephrotoxicity and electrolyte disturbances (e.g., hypokalemia and hypomagnesemia). Moreover, the co-administration of nephrotoxic drugs may augment the toxicity associated with amphotericin B. Therefore, this review aimed to provide a comprehensive overview of the management of oral and esophageal candidiasis from the viewpoint of clinical pharmacology, with a particular focus on drug–drug interactions and adverse effect profiles.

## 1. Introduction

Candida is a yeast that resides on the surface epithelium of the oral cavity in healthy individuals [1]. There are over one hundred *Candida* spp., including *C. albicans*, *C. glabrata*, *C. parapsilosis*, and *C. tropicalis* [2], which are causal pathogens of human mucosal and invasive infections [3]. *C. albicans* is a major pathogen responsible for oral and esophageal candidiasis [4,5]. *C. albicans* is the most common cause of oropharyngeal disease (approximately 70–80% of isolated cases); other causes include non-albicans species or mixed infections [3]. This review focuses on *C. albicans* and highlights the contributions of non-albicans species to disease patterns relevant to dental practice. *C. albicans* has a prevalence of approximately 30–50% in the oral cavity of healthy individuals, 50–65% in denture wearers, 65–90% in long-term hospitalized patients, and up to 90–95% in immunocompromised hosts [1], highlighting its role as an opportunistic pathogen. When immune function is compromised, *Candida* can colonize and proliferate in the oral and esophageal mucosa, leading to fungal invasion and the development of characteristic white plaques with underlying erythema [6]. Oral candidiasis commonly presents as pseudomembranous, erythematous (atrophic), chronic hyperplastic, denture stomatitis, or angular cheilitis [7]. Clinical diagnosis may be supported by KOH microscopy and culture (e.g., chromogenic media), with biopsy for hyperplastic lesions to exclude leukoplakia/dysplasia [8,9].

Oral and esophageal candidiasis typically occurs in immunocompromised individuals, such as those with malignancies, solid organ transplants, HIV infection, or those receiving cytotoxic chemotherapy and immunosuppressive agents (e.g., corticosteroids) [10,11,12,13,14]. Thus, individuals with poor oral condition, including those with dental prostheses or hyposalivation caused by radiation therapy or Sjögren’s syndrome, are at high risk of oral and esophageal candidiasis [15,16,17,18]. Pooled estimates suggest that the prevalence of oropharyngeal candidiasis is approximately 50% in high-risk cohorts, such as people with HIV, with *C. albicans* being the most frequently isolated species [19]. Upper endoscopy with histology or brush cytology is used to confirm esophageal candidiasis [5]. *C. albicans* is also the leading etiology for esophageal disease; upper endoscopy with histology and brushing is the diagnostic standard and helps distinguish Candida esophagitis from cytomegalovirus and herpes simplex virus infection, pill esophagitis, reflux esophagitis, and eosinophilic esophagitis [5]. Although the prognosis of oral and esophageal candidiasis is generally favorable, severe cases may result in systemic dissemination, especially in immunocompromised hosts [20,21]. Additionally, candidemia is associated with high mortality and morbidity due to complications such as endocarditis and ocular candidiasis [22]. A previous study reported that approximately 20% of patients experienced recurrence, often involving different *Candida* species from those isolated during the initial episode [23]. Therefore, optimal pharmacotherapy is essential for effective treatment.

However, considerable interindividual variability exists in the response to antifungal therapy, stemming from genetic and environmental factors (e.g., organ dysfunction, gene polymorphisms, and drug–drug interactions) [24,25,26]. Consequently, drug–drug interactions can complicate treatment, leading to therapeutic failure or unforeseen toxicity. Although previous reviews have focused on drug selection, only a few have comprehensively summarized pharmacokinetics, pharmacodynamics, and drug–drug interactions.

Therefore, this review aims to provide a comprehensive overview of drug–drug interactions and toxicity in oral and esophageal candidiasis, with a focus on clinical pharmacotherapy.

## 2. Oral Health Care

Effective management of oral candidiasis requires a holistic strategy that extends beyond routine oral hygiene. Oral health care refers to the comprehensive management of the oral cavity as a functional system including daily hygiene, maintenance of oral function, and targeted reduction in local risk factors. Rather than serving merely as an adjunct to antifungal pharmacotherapy, oral health care forms the foundation of treatment. It reduces the Candida burden and disrupts biofilms, which are intrinsically drug-tolerant, thereby enhancing the clinical response [27,28]. These measures are applicable across clinical forms of oral candidiasis and denture-related disease, in line with guidelines [29].

The central pillar of oral health care is the mechanical disruption and removal of biofilms and dental plaques. Dentate patients should brush their teeth twice daily, followed by interdental cleaning with floss or interdental brushes once daily. Routine cleansing of mucosal surfaces prone to Candida colonization, particularly the tongue dorsum—which serves as a major reservoir for Candida—is also recommended. Edentulous patients or those with compromised mucosal integrity such as those undergoing chemotherapy, require gentler cleaning techniques [30]. This may involve the use of soft-bristled toothbrushes, sponge swabs, or sterile gauze to clean the mucosa, palate, and tongue without causing trauma; however, the efficacy of plaque removal is relatively low [31].

Denture hygiene is another critical aspect of oral health care, as poorly maintained or long-term prostheses are established risk factors for denture stomatitis, which is a common manifestation of oral candidiasis [15]. An evidence-based regimen suggests combining daily mechanical cleaning with a non-abrasive cleanser and brief chemical disinfection (e.g., hypochlorite immersion). For acrylic appliances, short immersion in approximately 0.5% dilute sodium hypochlorite for no more than 10 min is recommended, while hypochlorite should be avoided for metal-containing dentures due to corrosion risk [32,33]. Additionally, nocturnal wear increases oral colonization and is associated with adverse outcomes in older adults [15]. Therefore, patients should be instructed to remove dentures overnight, which facilitates mucosal recovery and reduces exposure to fungal reservoirs.

Xerostomia (dry mouth) is a common and clinically significant cause of oral candidiasis. Reduced salivary flow compromises self-cleansing and diminishes key antimicrobial constituents, including mucins, histatins, lysozymes, lactoferrin, and secretory IgA, thereby favoring Candida adherence and persistence [34,35,36,37]. Moreover, acidic salivary conditions and a relatively closed, low-saliva environment at the denture–mucosa interface promote Candida growth and virulence; low salivary pH has been repeatedly linked to higher Candida carriage in denture wearers [38,39]. Overnight wearing of dentures exacerbates this condition, promoting early Candida adherence and increasing the risk of denture-related candidiasis [40].

Recommended management strategies include maintaining adequate fluid intake, stimulating saliva production with sugar-free gum or candy, and using saliva substitutes [29,41]. The management of xerostomia requires a coordinated multidisciplinary approach because it often arises from multiple causes, including systemic diseases, medications, oral hypofunction, and chemotherapy. Consequently, collaboration among physicians, dentists, and pharmacists is essential for effective intervention [42,43,44].

## 3. Clinical Guidelines for the Treatment of Oral and Esophageal Candidiasis

Therapeutic options for oral and esophageal candidiasis are summarized in Table 1 and Table 2, and treatment is determined by disease severity and site of infection [29]. In pregnant women, intravenous amphotericin B is an alternative, as systemic azole antifungals are contraindicated in the first trimester owing to teratogenicity. In such cases, local administration is preferred.

### 3.1. Oral Candidiasis

First-line treatment for mild oral candidiasis involves the topical administration of clotrimazole troches, miconazole mucoadhesive buccal tablets, nystatin suspensions, or pastilles [45,46,47,48]. Oral fluconazole is recommended in cases of moderate-to-severe disease or unresponsiveness to first-line treatment [49,50,51]. When fluconazole treatment fails, increased dose of fluconazole or other azole antifungals, including itraconazole, posaconazole, and voriconazole are administered [52,53,54]. Recommended treatment duration for patients without serious comorbidities is 7–14 days, with further extension for 14–28 days in patients with refractory disease [29].

### 3.2. Esophageal Candidiasis

Systemic fluconazole is recommended as the initial treatment of esophageal candidiasis, regardless of the severity [29,55]. For patients who cannot tolerate fluconazole or in whom it is contraindicated, intravenous echinocandins or amphotericin B are recommended as alternative initial therapies. In refractory cases, options include dose escalation of fluconazole or switching to another antifungal agent (itraconazole, posaconazole, voriconazole, intravenous echinocandins, or liposomal amphotericin B). The recommended duration of therapy is 14–21 days.

## 4. Pharmacological Mechanism

**Figure 1:**
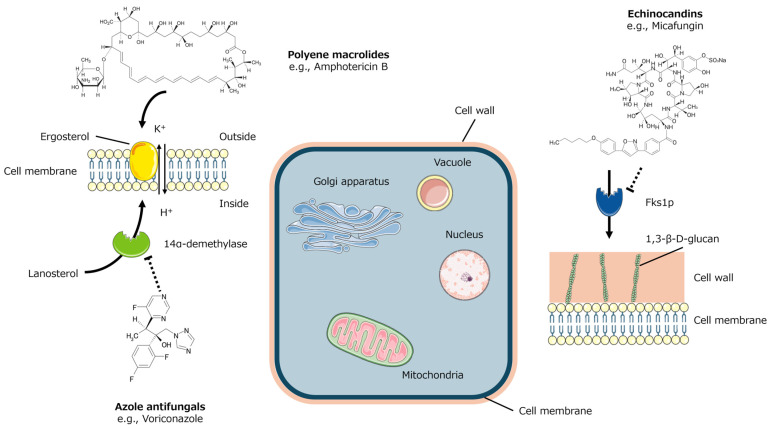
Mechanism of action of individual antifungal agents. Prepared using previously published data [56,57,58,59,60,61].

The pharmacological actions of the individual antifungal agents are shown in Figure 1. Notably, azole antifungals and echinocandins exhibit selective toxicity toward Candida because their target molecules are specific to fungi and are not found in host cells.

Amphotericin B, a polyene macrolide, specifically binds to ergosterol in the fungal cell membrane and forms pores that allow the passage of protons and monovalent cations such as potassium through increased membrane permeability [56,57,58]. This leads to membrane depolarization, affecting fungal and host cells. A liposomal formulation of amphotericin B has been developed and is widely used to reduce toxicity while maintaining antifungal efficacy [62]. Topical nystatin, which is poorly absorbed systemically, has minimal adverse effects and is particularly useful for the reducing colonization of *Candida* on the tongue dorsum, a major reservoir of this organism [63].

In contrast, azole antifungals, including triazoles and imidazoles, inhibit a key enzyme 14-α-demethylase (CYP51A1), which catalyzes the conversion of lanosterol to ergosterol, an essential component of fungal membranes [59,60]. Inhibition of this enzyme results in the accumulation of toxic sterol intermediates. Consequently, azole antifungals disrupt membrane integrity and function, thereby hindering fungal growth.

The fungal cell wall consists of several polysaccharides, including 1,3-β-D-glucan, 1,4-β-D-glucan, 1,6-β-D-glucan, and diverse glycoproteins [64]. Accordingly, echinocandins, a class of cyclic hexapeptides, noncompetitively block the Fks1p subunit of the enzyme responsible for synthesizing 1,3-β-D-glucan, which is a specific component of the fungal cell wall [61]. This action weakens the fungal cell wall, leading to osmotic instability and cell lysis.

## 5. Clinical Pharmacokinetics

Antifungals used to treat candidiasis exhibit diverse pharmacokinetic profiles [65,66,67], and determining their ability to reach infection sites is a primary clinical concern. Amphotericin B has poor oral bioavailability (approximately 0%) and remains confined to the gastrointestinal lumen when administered orally [68]. In contrast to conventional amphotericin B, liposomal amphotericin B exhibits lower tissue permeability [69,70]. Amphotericin B is not metabolized by cytochrome P450 (CYP) enzymes and is primarily eliminated via renal excretion [70]. In contrast, liposomal amphotericin B exhibits nonlinear pharmacokinetics [58] because lipid carriers are relatively large particles that restrict the diffusion of drugs into non-pathological tissues, while preferentially accumulating in infected tissues due to increased vascular permeability.

Azole antifungals, except fluconazole, are substrates of CYP enzymes and drug transporters [71]. For instance, CYP3A4 mediates the hepatic metabolism of itraconazole to its active metabolite, 14-hydroxyitraconazole, whereas plasma esterases convert isavuconazonium to its active moiety, isavuconazole [72,73]. Voriconazole is primarily metabolized by both CYP2C19 and CYP3A4. Its nonlinear pharmacokinetics results from the saturation of CYP2C19, whose activity varies substantially due to genetic polymorphisms [74,75]. In contrast, posaconazole predominantly undergoes metabolism via uridine diphosphate glucuronosyltransferase and is carried via P-glycoprotein (P-gp) to efflux from the cell into the lumen [76]. Fluconazole is largely eliminated via glomerular filtration following tubular reabsorption [74].

Caspofungin, an echinocandin, is degraded by hydrolysis and N-acetylation, and its metabolites are mainly excreted in bile and feces [77]. Micafungin is metabolized by sulfatase into the catechol form, which is further metabolized by catechol O-methyltransferase into the methoxy form and finally undergoes non-enzymatic ring-opening to form a methoxy derivative [78].

## 6. Toxicity

Systemic administration of amphotericin B causes infusion-related reactions, typically manifesting as fever and chills [68] (Table 3). These reactions are believed to result from the increased prostaglandin E2 synthesis [79]. Premedication is recommended to prevent infusion-related reactions [80]. For instance, premedication with corticosteroids can mitigate these symptoms, unlike non-steroidal anti-inflammatory drugs and acetaminophen [81].

Nephrotoxicity, a dose-dependent and generally reversible adverse effect of amphotericin B, often limits its systemic administration [68,82,83]. The drug binds to cholesterol in host cell membranes, increasing membrane permeability in the renal vasculature. Additionally, amphotericin B increases the permeability of the macula densa cells, which sense sodium chloride delivery. This leads to the activation of the tubuloglomerular feedback mechanism, afferent arteriolar vasoconstriction, and a subsequent reduction in the glomerular filtration rate. Altered membrane permeability subsequently results in electrolyte disturbances, including the loss of potassium and magnesium [68]. Indeed, sodium enters distal tubular cells via amphotericin B–induced pores, followed by potassium efflux [84,85].

Long-term use of azole antifungals can result in hepatotoxicity and hormone-related adverse effects (e.g., gynecomastia and adrenal insufficiency) [86]. Hepatotoxicity associated with azole antifungals manifests as hepatocellular and cholestatic patterns [86]. However, the mechanisms underlying this hepatotoxicity are unclear. Nevertheless, the blood concentration of voriconazole is useful for identifying populations at high risk of hepatotoxicity, especially among Asian populations [87,88].

Azole antifungals block lanosterol 14-α-demethylase, which is involved in the production of ergosterol [89]. Azoles can also inhibit mammalian CYP enzymes, which are involved in steroidogenesis, leading to endocrine-related adverse effects such as gynecomastia, adrenal insufficiency, and pseudo-hyperaldosteronism [90]. They are also known to prolong QT interval; however, the exact mechanism is not fully understood. Several azoles also inhibit the human Ether-à-go-go–Related Gene (hERG) potassium channel, which is involved in cardiac repolarization [91]. Therefore, drug interactions are a notable concern, as azoles can inhibit multiple CYP enzymes and transporters, resulting in elevated plasma concentrations of co-administered drugs with a potency of QT prolongation [92]. However, a previous study reported that isavuconazole shortened QT intervals in a dose-dependent fashion [93]. Moreover, voriconazole is associated with visual disturbances (e.g., photopsia) and concentration-dependent central nervous system side effects, while itraconazole may cause gastrointestinal symptoms, partly due to its additive agent with β-cyclodextrin [94,95].

Echinocandins exhibit better tolerability than amphotericin B and azoles [96]. The most common adverse effects of echinocandins include infusion-related reactions such as rashes and flushing [97]. Although fever is particularly common with caspofungin, it may be alleviated by reducing the infusion rate [98]. Mild elevations in hepatic aminotransferases and alkaline phosphatase can also occur due to caspofungin but are typically asymptomatic and reversible [99,100].

## 7. Drug–Drug Interaction

Drug–drug interactions can occur through different mechanisms, resulting from either pharmacokinetics or pharmacodynamics. Antifungal agents interact with a variety of drugs. Some common antifungal drug–drug interactions are discussed below.

### 7.1. Pharmacokinetic Interaction

**Figure 2:**
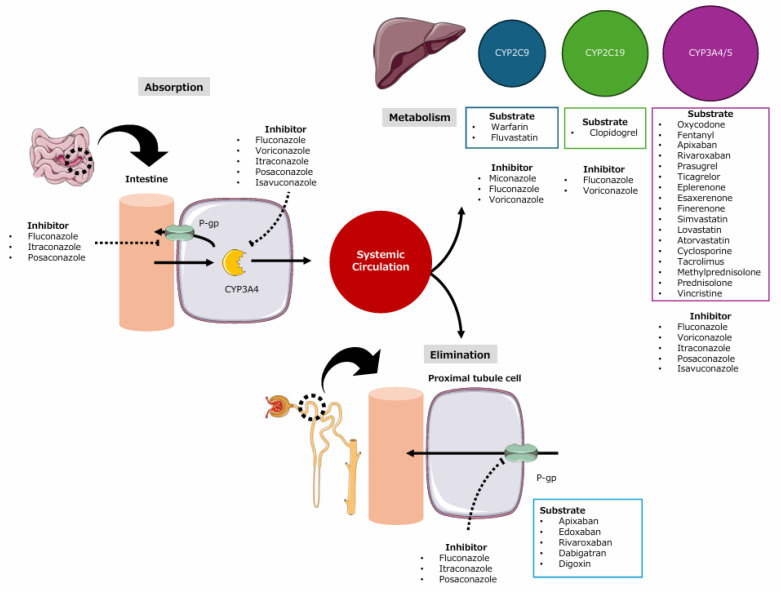
Summary of drug–drug interaction regarding CYP isoforms and P-gp. Abbreviations: CYP: cytochrome P450 and P-gp: P-glycoprotein. Each circle represents an abundance of CYP enzymes. Prepared using previously published data [74,101,102,103,104].

Pharmacokinetic interactions arise when one drug alters the pharmacokinetic processes of another drug, including absorption, distribution, metabolism, and excretion. Most clinically relevant interactions involve drug metabolizing enzymes and transporters such as CYP and P-gp. The mechanism of drug–drug interactions is illustrated in Figure 2. CYP3A4 is the most abundant drug-metabolizing enzyme in the gut and liver [101,102,103], with inter-individual variability largely explained by genetic polymorphisms [102]. In contrast, P-gp expressed on the apical membrane exports substrates out of the cell [104].

Additionally, drug–drug–gene interactions should be considered when planning a treatment strategy [105,106]. Genetic variations exist in drug-metabolizing enzymes and transporters. Thus, the extent of drug interactions varies according to genetic polymorphisms, even when patients receive the same victim–perpetrator combination. For example, a common interaction between tacrolimus and voriconazole results from the inhibition of CYP3A4 metabolism [106,107,108,109]. This effect is more pronounced in carriers of CYP2C19 loss-of-function alleles, since CYP2C19 polymorphisms strongly influence voriconazole clearance [110,111].

Azole antifungals are particularly problematic in clinical practice because they inhibit multiple CYP isoenzymes and P-gp [74,112,113]. The addition of azole antifungals leads to a sustained increase in the co-administered victim drug concentrations. Unlike other antifungal classes, since azoles are often administered for weeks, even moderate inhibition can accumulate over time. Consequently, azoles are recognized as “high-risk perpetrators” of drug–drug interactions and require therapeutic drug monitoring or careful dose adjustment as needed.

Although azole antifungals inhibit CYP enzymes and transporters, their targets vary among the individual agents (Table 4). Itraconazole is a strong inhibitor of CYP3A4 and P-gp [114,115]. Additionally, a metabolite of itraconazole (14-hydroxyitraconazole) is an equally potent CYP3A4 inhibitor [115]. Meanwhile, voriconazole is recognized as a substrate and inhibitor of CYP2C9, CYP2C19, and, to a lesser extent, CYP3A4 [67]. The extent of this interaction depends on the plasma concentration of voriconazole. The extent of interaction depends on plasma voriconazole concentration; tacrolimus exposure increases markedly when voriconazole levels exceed 2.0 µg/mL [116]. Fluconazole is a moderate inhibitor of CYP2C9, CYP2C19, and CYP3A4 [117].

#### 7.1.1. Opioids

The pharmacokinetics of opioids, such as oxycodone and fentanyl, involve CYP3A4 metabolism [118]. Consequently, azole antifungals interfere with oxycodone metabolism [119]. The co-administration of voriconazole increases the risk of oxycodone-induced somnolence [120]. In contrast, the inhibition of CYP3A4 slightly affects fentanyl when administered intravenously [121]. This is because fentanyl clearance depends primarily on hepatic blood flow rather than CYP activity. Thus, the administration route produces variations in drug–drug interactions between opioids and azole antifungals.

#### 7.1.2. Antithrombotic Drugs

Warfarin is a racemic mixture of R and S isomers [122]. S-warfarin is more than five times as potent as R-warfarin [123,124]. S-warfarin mainly undergoes the metabolism of CYP2C9 [125,126]. Thus, the CYP2C9 inhibitor fluconazole inhibits warfarin metabolism and increases bleeding risk [127,128]. Similarly, a clear elevation in the prothrombin time international normalized ratio (PT-INR) is observed when voriconazole is used with warfarin [129]. Miconazole, a topical formulation, has been reported to interact with the CYP2C9 metabolism of warfarin, and thereby elevates PT-INR [130,131,132]. This is attributed to the absorption of miconazole by the gut. Dose optimization is a valuable approach for managing bleeding risk when warfarin is administered.

All direct oral anticoagulants are substrates of P-gp in the gut and proximal tubules [133]. Accordingly, several azole antifungals that inhibit P-gp are associated with increased blood concentrations of direct oral anticoagulants [134]. A study of the drug interactions between rivaroxaban and fluconazole suggested a notable impact on the pharmacokinetics of rivaroxaban. Moreover, this interaction is harmful when rivaroxaban is co-administered with fluconazole in addition to cyclosporine (inhibitors of CYP3A4 and P-gp) [135]. The contribution of CYP3A4 varies among DOACs; it is particularly important for apixaban and rivaroxaban. Co-administration with strong CYP3A4 inhibitors or inducers should be avoided in these cases [136,137].

Clopidogrel undergoes a 2-step conversion with an intermediate active metabolite via mainly CYP2C19 [138]. Prasugrel is activated by CYP3A4 and 2B6 [139]. Ketoconazole decreases the active metabolite formation of clopidogrel and attenuates the pharmacodynamic effect on platelet function but does not affect the pharmacokinetics of prasugrel [140]. However, ticagrelor, a pure CYP3A4 substrate, is not recommended when a strong CYP3A4 inhibitor (itraconazole) is administered [141].

#### 7.1.3. Cardiovascular Drugs

The primary elimination route of digoxin involves glomerular filtration and secretion via P-gp [142]. In addition to amiodarone, the inhibition of P-gp by itraconazole and posaconazole alters the bioavailability and renal clearance, increasing the risk of digoxin intoxication [143,144,145,146].

CYP3A4 is essential for the metabolism of several mineralocorticoid receptor antagonists, including eplerenone, esaxerenone, and finerenone [147,148,149]. Close monitoring should be performed for the hyperkalemic risk of mineralocorticoid receptor antagonists co-administered with CYP3A4 inhibitors, including azole antifungals [112]. Older age increases the risk of hyperkalemia when mineralocorticoid receptor antagonists and CYP3A4 inhibitor simultaneously [150].

The pharmacokinetic profiles of statins differ in terms of drug transporters and metabolism. Hepatic metabolism occurs primarily through CYP3A4 for simvastatin, lovastatin, and atorvastatin, whereas fluvastatin is metabolized mainly through CYP2C9 and pitavastatin and rosuvastatin are converted through to lesser extent, CYP2C9 [151,152]. The CYP3A4 inhibitor dramatically increases statins metabolized by CYP3A4 (e.g., simvastatin), whereas fluconazole interacts with fluvastatin via the CYP2C9 interaction [153]. Hepatic uptake of statins is mediated by organic anion-transporting polypeptides 1B1 and 1B3, but not P-gp [152]. Thus, the switch from simvastatin to pravastatin can be ignored when co-administrating with itraconazole [154].

#### 7.1.4. Immunosuppressant Drugs

An immunosuppressive regimen of calcineurin inhibitors (e.g., tacrolimus and cyclosporine) and glucocorticoids is often used to treat autoimmune diseases and transplantation.

There is large variability in the pharmacokinetics of calcineurin inhibitors, suggesting their utility in therapeutic drug monitoring [155,156,157]. Cyclosporine is a substrate of CYP3A4 and P-gp [89]. A randomized study identified a slow increase in cyclosporine concentration during the 2 weeks following fluconazole administration [158]. In particular, close monitoring of drug concentration is recommended for fluconazole doses above 200 mg/day [159]. Posaconazole has a greater impact on the pharmacokinetics of cyclosporine than fluconazole [160]. Tacrolimus is primarily metabolized by CYP3A5, and to a lesser extent by CYP3A4 [161]. Numerous differences exist in the effects of azole antifungals on tacrolimus pharmacokinetics [162]. The interaction between tacrolimus and azole antifungals was blunted by CYP3A5 expressor [163]. Moreover, drug–drug interactions between tacrolimus and azole antifungals differ depending on the administration route [164]. This is because intravenous administration of tacrolimus is not relevant to the metabolism of CYP3A4 in the intestine.

Co-administration of azole antifungals blocks CYP3A4, which potentially increases the exposure to both methylprednisolone and prednisolone. However, itraconazole causes no change in the pharmacokinetic profile of prednisolone despite notable adrenal suppression by methylprednisolone [165]. Isavuconazole did not affect the exposure to prednisolone [166]. Thus, the combination of glucocorticoids and azole antifungals affects the magnitude of drug–drug interactions.

#### 7.1.5. Chemotherapeutic Drugs

Several studies have suggested that the interactions between drugs and azole antifungals drive the development of peripheral neuropathy caused by vinca alkaloids. Azole antifungals inhibit CYP3A4 metabolism of vincristine and increase the risk of toxicity, including peripheral neuropathy and secondary constipation, due to neuropathy in hematologic malignancies [167]. In addition, itraconazole inhibits CYP3A4 and P-gp, further contributing to the elevation of intracellular concentrations in nerve cells [168]. In contrast, fluconazole has less potential to increase the risk of neurotoxicity than itraconazole and voriconazole [169].

Most kinase inhibitors are metabolized by CYP3A4 [170]. Co-administration of azole antifungals predisposes patients to drug–drug interactions with tyrosine kinase inhibitors [171]. Therefore, individual doses should be personalized based on in-depth discussions among the medical staff to avoid undesirable adverse events.

### 7.2. Pharmacodynamic Interaction

Pharmacodynamic interactions occur when the pharmacological effects of a drug are altered by another co-administered drug. This interaction is generally classified as synergistic, additive, or antagonistic, according to the combination therapy. In clinical practice, the additive effects pose significant risks, particularly when they lead to unintended toxicity.

#### Nephrotoxicity and Electrolyte Disorder

Drug-induced nephrotoxicity involves multiple mechanisms [172]. For instance, nephrotoxicity with amphotericin B is exacerbated when co-administered with other nephrotoxic drugs, such as calcineurin inhibitors and antihypertensive drugs (angiotensin-converting enzyme inhibitors and angiotensin II receptor blockers) [173,174,175].

Likewise, additive effects on electrolyte depletion are considered when amphotericin B is combined with drugs that promote the urinary excretion of electrolytes. Loop and thiazide diuretics increase the urinary excretion of potassium and magnesium by inhibiting sodium-potassium-chloride and sodium-chloride symporters, respectively [176]. Glycyrrhizin blocks 11β-hydroxysteroid dehydrogenase 2 that converts cortisol (active form) to cortisone (inactive form), thereby elevating cortisol level [177]. Consequently, cortisol acts on mineralocorticoid receptors and enhances renal potassium excretion. In addition, older age is a risk factor for Kampo medicine-induced hypokalemia [178]. Fludrocortisone also binds to mineralocorticoid receptors and thereby increases the risk of hypokalemia [179].

Epidermal growth factor receptor (EGFR) plays a key role in renal tubular signaling [180]. The anti-epidermal growth factor receptor (EGFR) monoclonal antibodies cetuximab and panitumumab, which have relatively long half-lives, target the EGFR cascade and cause hypomagnesemia [181]. This mechanism impairs the expression of channel transient receptor potential M6 that reabsorbs magnesium in the distal nephron [182]. Therefore, the combination of amphotericin B and anti-EGFR monoclonal antibodies may increase the risk of hypomagnesemia. Furthermore, magnesium deficiency often leads to refractory hypokalemia because of the release of magnesium-mediated inhibition of ROMK, which promotes potassium secretion into urinary lumen [183,184].

Additional pharmacodynamic concerns include additive QT prolongation and hormone-related effects of azole antifungals. However, these largely overlap with the intrinsic toxic profiles described earlier.

## 8. Discussion

The management of oral and esophageal candidiasis extends beyond symptom relief to influence long-term prognosis, particularly in immunocompromised patients. Although prognosis is generally favorable with appropriate treatment, recurrence occurs in approximately 20% of patients, and systemic dissemination can be life-threatening. Therefore, the pharmacological issues described in this review, such as drug–drug interactions, adverse effect profiles, and interindividual variability in drug metabolism, are critical determinants of treatment outcomes.

For example, drug–drug interactions with antifungals may lead to supratherapeutic drug concentrations or toxicity by immunosuppressants, anticoagulants, or chemotherapeutic agents, and increase the risk of treatment failure and Candida recurrence. Similarly, the impact of CYP2C19 polymorphisms on voriconazole metabolism underscores how genetic variability can affect both efficacy and safety. These findings suggest that antifungal therapy should be individualized, taking into account comorbidities, concomitant medications, and genetic background, to improve prognosis and prevent refractory or disseminated infections.

Antifungal resistance is a significant factor affecting prognosis. Repeated or prolonged azole exposure, particularly in immunocompromised hosts, has contributed to the increasing prevalence of fluconazole-resistant species, such as *C. glabrata*, and intrinsically resistant species, such as *C. krusei* [185]. Resistance not only compromises the efficacy of first-line agents but also necessitates the use of broader spectrum or more toxic alternatives, which may increase the risk of drug interaction and adverse events. Thus, antifungal susceptibility patterns and timely adjustments to therapy are essential for preventing treatment failure and improving long-term outcomes.

The effective management of oral candidiasis requires a multidisciplinary collaborative approach. Physicians assess and manage systemic risk factors, such as immune deficiency and diabetes. They also guide decisions regarding systemic antifungal therapy. Dental professionals confirm the diagnosis through intraoral examination and address local predisposing factors such as ill-fitting dentures and xerostomia. Mechanical disruption of Candida biofilms via professional denture cleaning and oral care is essential to enhance antifungal efficacy [186]. Pharmacists play a key role in ensuring the safe and effective use of antifungal agents, especially azole antifungals, which can cause complex drug interactions [187]. Prescription verification, drug–drug interaction monitoring, and patient counseling help optimize therapeutic outcomes.

In addition to the pharmacological considerations discussed above, emerging antifungal resistance and the increasing complexity of polypharmacy highlight the need for more individualized treatment strategies. Despite the availability of guidelines, limited attention has been paid to pharmacogenetic variability and real-world drug–drug interactions, particularly in elderly and immunocompromised populations. Future research should integrate pharmacokinetic–pharmacodynamic modeling, therapeutic drug monitoring, and pharmacogenomic testing to optimize antifungal exposure and minimize toxicity. Moreover, interprofessional collaboration among physicians, dentists, and pharmacists is critical not only for preventing recurrent or refractory infections but also for developing personalized antifungal stewardship frameworks. Such efforts are expected to bridge the current gap between pharmacological knowledge and clinical implementation.

Effective management of oral candidiasis requires both appropriate antifungal selection and seamless interdisciplinary collaboration among physicians, dentists, and pharmacists.

## 9. Conclusions

The optimal management of oral and esophageal candidiasis requires effective pharmacological therapy. Appropriate drug selection, careful monitoring of drug–drug interactions, and attention to toxicity are essential for therapeutic efficacy maximization and toxicity minimization. In combination with interindividual variability, including genetic polymorphisms that affect drug metabolism and transport, this underscores the need for personalized treatment strategies.

Although oral health care and multidisciplinary collaboration are necessary to support the treatment of oral and esophageal candidiasis, the central focus should be on the safe and effective use of antifungal agents. Future research should clarify how pharmacogenomic testing and therapeutic drug monitoring can be integrated into routine care to further improve prognosis in patients with oral and esophageal candidiasis.

## Figures and Tables

**Table 1:** Therapeutic options for oral candidiasis.

Drug	Formulation	Route	Dose	Frequency
First line (mild)				
Clotrimazole	Troches	Topical (oral cavity)	10 mg	5 times/day
Nystatin	Suspension Pastilles	Topical (oral cavity)	400,000–600,000 U (suspension) 1 pastille (pastilles)	4 times/day
Miconazole	Mucoadhesive buccal tablet	Topical (buccal)	50 mg	Once daily
First line (Moderate to Severe)				
Fluconazole	Tablet/capsule	Oral	100–200 mg (Day 1: 200 mg)	Once daily
Alternative option				
Itraconazole	Oral solution	Oral	200 mg	Once daily
Posaconazole	Oral suspension	Oral	400 mg	Twice daily for 3 days, then once daily
Voriconazole	Tablet	Oral	200 mg	Twice daily

The maximum recommended daily dose of fluconazole is 400 mg.

**Table 2:** Therapeutic options for esophageal candidiasis.

Drug	Formulation	Route	Dose	Frequency
First line				
Fluconazole	Tablet/capsule, IV solution	Oral/IV	200–400 mg (Day 1: 400 mg)	Once daily
Second line				
Itraconazole	Oral solution	Oral	200 mg	Once daily
Posaconazole	Oral suspension DR tablet	Oral	400 mg (suspension) 300 mg (tablet)	Once or twice daily
Voriconazole	Tablet, Injection	Oral/IV	200 mg	Twice daily
Micafungin	Injection	IV	150 mg	Once daily
Amphotericin B deoxycholate	Injection	IV	3 mg/kg	Once daily
Liposomal amphotericin B	Injection	IV	3 mg/kg	Once daily

Abbreviations: IV: intravenous. The maximum recommended daily dose of fluconazole is 800 mg.

**Table 3:** Major adverse effects of systemic antifungal agents.

Drug Class	Adverse Events	Comment
Polyene macrolides (Amphotericin B)	Infusion reaction Nephrotoxicity, hypokalemia, hypomagnesemia	Liposomal formulations reduce toxicity
Azoles	Hepatotoxicity Gynecomastia, adrenal suppression QT prolongation (except isavuconazole)	Voriconazole; CNS toxicity and visual disturbance
Echinocandins	Infusion reaction Hepatotoxicity	Generally tolerated

Abbreviations: CNS: central nervous system.

**Table 4:** Inhibition of azole antifungals with CYP enzymes and drug transporters.

	Fluconazole	Miconazole	Itraconazole	Voriconazole	Posaconazole	Isavuconazole
Substrate						
CYP2C9				Y		
CYP2C19				Y		
CYP3A4			Y	Y		Y
P-gp					Y	
Inhibitor						
CYP2C9	++	++		+		
CYP2C19	+++			++		
CYP3A4	++		+++	+++	+++	++
P-gp	+		+		+	

Abbreviations: CYP: cytochrome P450, P-gp: P-glycoprotein. +: Mild, ++: Moderate, +++: Severe, Y: yes.

## Data Availability

Data sharing is not applicable.

## Abbreviations

14-α demethylase: CYP51A1; cytochrome P450: CYP; P-glycoprotein: P-gp; Ether-à-go-go–Related Gene: hERG; prothrombin time international normalized ratio: PT-INR; Epidermal growth factor receptor: EGFR.
